# Structural MRI correlates of cognitive and neuropsychiatric symptoms in Long COVID: a pilot study

**DOI:** 10.3389/fpsyt.2024.1412020

**Published:** 2024-12-04

**Authors:** Shantanu H. Joshi, Prabha Siddarth, Helen Lavretsky

**Affiliations:** ^1^ Department of Neurology, David Geffen School of Medicine, University of California, Los Angeles, Los Angeles, CA, United States; ^2^ Department of Bioengineering, University of California, Los Angeles, Los Angeles, CA, United States; ^3^ Department of Psychiatry and Biobehavioral Sciences, David Geffen School of Medicine, University of California, Los Angeles, Los Angeles, CA, United States

**Keywords:** long COVID, structural MRI, brain, cortical thickness, cingulate gyrus, gray matter volume

## Abstract

Approximately 7% of COVID-19 patients (1.3% children) have exhibited symptoms of post-acute sequelae of SARS-CoV-2 infection (PASC), or Long COVID, and 20% of those present with neuropsychiatric symptoms. While a large number of MRI-based neuroimaging studies in this population have shown cortical atrophy in terms of gray matter volume and cortical thickness in patients, there is a growing body of work showing brain volume enlargements or thickness increases in patients compared to COVID negative controls. To investigate this further, we used structural magnetic resonance imaging (MRI) to examine differences in gray matter thickness for the cortical limbic and the dorsolateral prefrontal cortical regions between patients with Long COVID and healthy controls. Results showed increased cortical thickness in the caudal anterior, isthmus, and the posterior cingulate gyrus as well as the rostral middle frontal gyrus respectively along with higher gray matter volume in the posterior cingulate and the isthmus cingulate in patients with Long COVID. Cortical thickness and gray matter volumes for regions of interest (ROIs) were also associated with the severity measures, clinical dementia rating, and anxiety scores in the Long COVID group. Our findings provide supporting evidence for cortical hypertrophy in Long COVID.

## 1 Introduction

COVID-19 infection is a respiratory disease that took the world by storm after its emergence in December 2019 and was caused by the SARS-CoV-2 virus. Acute symptoms of SARS-CoV-2 infection can range in severity, but common symptoms include cough, fever, chills, etc. or in some more severe cases, kidney failure, nervous system problems, and even death. As of April 2024, there have been 775 million confirmed cases of COVID-19 and over 7 million deaths worldwide as per the World Health Organization (WHO) (1). Additionally, since the emergence of the pandemic, a phenomenon known as ‘Long COVID’ or Post-acute sequelae of COVID-19 (PASC) has affected multiple organs with an increasing risk with every subsequent re-infection (2). Although the Long COVID syndrome is still being studied, currently it is defined by the presence of persistent symptoms and/or delayed long-term complications of SARS-CoV-2 infection beyond 4 weeks from the onset of symptoms (3). These ‘complications’ range from brain fog, cognitive impairment, or fatigue and also involve symptoms such as irregular menstruation and chest pains thus affecting almost every organ system. Neuropsychiatric symptoms such as fatigue, subjective cognitive impairment (“brain fog”), anxiety, and depression are the most common Long COVID symptoms (4, 5). Currently, it is estimated that around 7% of COVID-19 cases experience Long COVID (6–8), which may be underestimated due to reduced awareness of the symptoms and the disease course among patients and healthcare providers. The underlying mechanisms and causes of Long COVID are still unknown, but one study has documented one risk factor- high levels of cortisol predicted clinical and immunological manifestations of Long COVID (9). A recent meta-analysis (10) identified 19 studies, encompassing a total of 11,324 patients. Overall the rates of psychiatric symptoms in Long COVID were: fatigue (37%, 95% CI: 24%-50%), brain fog (32%, 9%-55%), memory issues (27%, 18%-36%), attention disorder (22%, 10%-34%), myalgia (18%, 4%-32%), anosmia (12%, 7%-17%), dysgeusia (11%, 4%-17%) and headache (10%, 1%-21%). neuropsychiatric conditions included sleep disturbances (31%, 18%-43%), anxiety (23%, 13%-33%) and depression (12%, 7%-21%). Neuropsychiatric symptoms substantially increased in prevalence over longer-term follow up. Patients who were hospitalized for Long -COVID had much higher rates of neuropsychiatric symptoms. Cohorts with >20% of patients admitted to the ICU during acute COVID-19 experienced higher prevalence of fatigue, anxiety, depression, and sleep disturbances than cohorts with <20% of ICU admission.

Studies have been largely conducted on older populations over the age of 50 as well as more severe cases of infection, compared to milder cases. Several reports detected neural changes and abnormalities after a COVID-19 diagnosis (11, 12). Brain imaging, specifically magnetic resonance imaging (MRI), is the primary technique used for a wealth of these studies to comprehend structural and functional changes in the brain. Douaud et al. investigated structural changes in the brain in UK Biobank in cases of COVID-19 (13). Their research focused primarily on differences post infection, with 141 days between scans. Cognitive decline measured through six cognitive tasks was seen to be higher on average in the experimental group, even after the exclusion of hospitalized patients. Additionally, structural changes included reduced global brain size, reduced thickness and contrast of gray matter in the parahippocampal gyrus and orbitofrontal cortex, and markers of tissue damage in the olfactory pathways. Even after excluding hospitalized patients, these differences were still observed (13). These changes were hypothesized to be related to the virus’ spread through olfactory pathways, although further research is needed. Limited research is available to characterize brain changes in Long COVID. Increased gray matter volume primarily in the limbic and olfactory systems in patients with Long COVID 8 months after infection has been previously reported (5). Additionally, voxel-based morphometry (VBM) analysis indicated larger gray matter volume in various areas of the brain including the hippocampus and the amygdala (5). Furthermore, subcortical nuclei and white matter differences were dependent on severity of infection and duration of recovery or rehabilitation (14). These results are conflicting with results of the COVID studies detailed above, with the primary wealth of literature surrounding COVID-19 suggesting a reduction in gray matter volume or thickness. These novel findings could potentially indicate potentially differing mechanisms of viral infection or compensatory mechanisms in case with Long COVID and those without.

**Figure 1:**
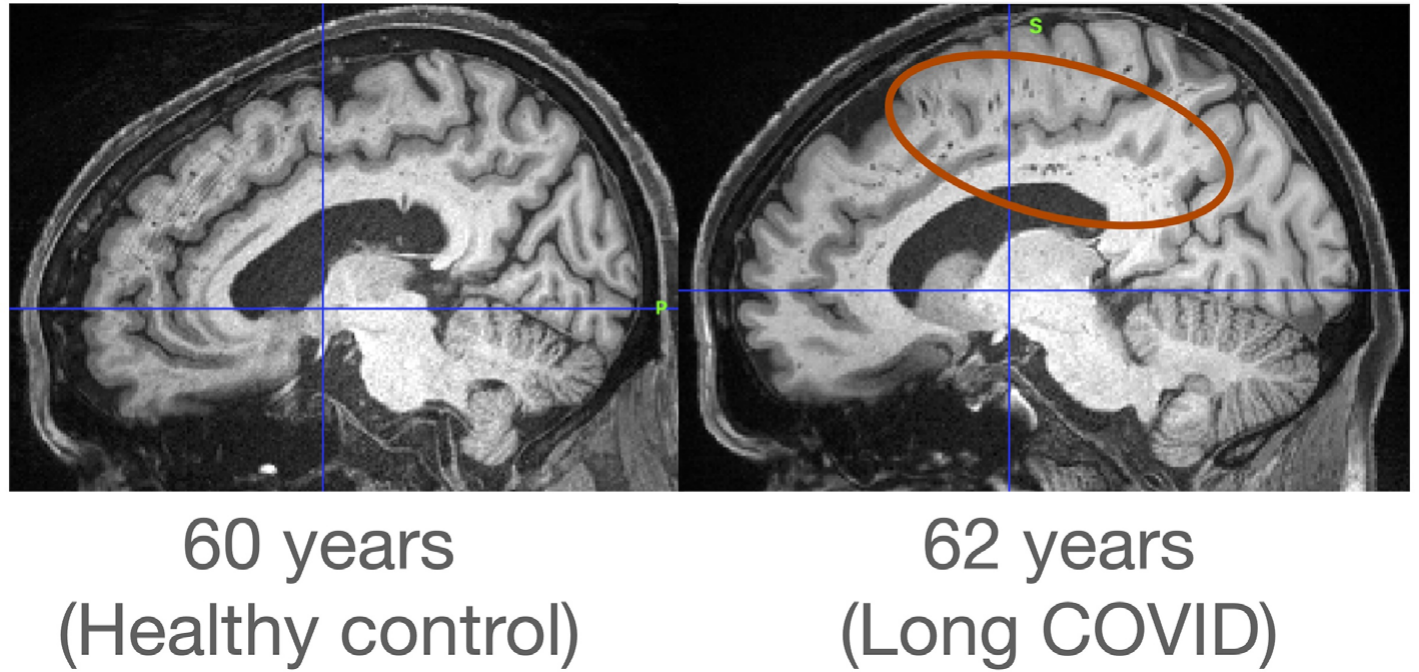
T1‐weighted MR images for a healthy control of age 60 years and a participant diagnosed with Long COVID of age 62 years. The red circle denotes white matter hyperintensities.

For illustrative purposes, **Figure 1** shows T1‐weighted MR images for a healthy control of age 60 years and a participant who was diagnosed with Long-COVID of age 62 years. The subject with Long-COVID showed white matter hyperintensities in the regions associated with the corona radiata.

In this study, we used structural MRI to investigate the anatomical brain differences between subjects with Long COVID and COVID-negative participants (COVID-) along with the associations between structural measures and the severity of chronic medical illness as well as anxiety scores. Since the focus is on Long COVID, we selected the dorsolateral prefrontal cortex (DLPFC) comprising the rostral middle frontal gyrus and the caudal middle frontal gyrus, and the cingulate gyrus (caudal anterior cingulate, posterior cingulate, and the isthmus cingulate), the entorhinal cortex, and the insular cortex. The DLPFC is primarily selected as it is implicated in cognitive function including working memory and executive function and has shown to be susceptible to an inflammatory immune response due to PASC (15, 16). Further, the DLPFC is also being investigated as a potential neuromodulatory target to relieve the symptom severity associated with PASC (17–19). Finally, the cingulate gyrus was selected as its role in emotion regulation and episodic memory processing is impaired in Long COVID (20, 21). The insula has been implicated in Long-COVID in several recent studies (5, 22). Finally, the entorhinal cortex was selected because cognitive impairments in Long-COVID participants show common features with those of Alzheimer’s disease and related dementias (23). Our study aims to investigate these regions, to map the structural changes and their associations with clinical measures in PASC.

## 2 Methods

### 2.1 Participants and clinical assessments

Participants for this study were recruited from the UCLA hospital and the broader Los Angeles community. They included 36 individuals (14 males and 22 females) ranging from ages 20 to 67 years. 28 of these participants who had received a COVID-19 diagnosis approximately one year prior of performing the study. The remaining 8 were COVID negative (no prior history of COVID) and were classified as COVID negative controls. Of the 28 COVID-19 diagnosed participants, 21 participants were scanned successfully, of which 15 subjects were deemed to have Long COVID (Long-COVID), while 6 subjects were previously diagnosed with COVID but did not exhibit any symptoms of Long COVID. Owing to the small sample size of the non-Long COVID participants, in this paper, we only focused on a comparative analysis between Long-COVID participants and COVID- controls. COVID-19 tests were not conducted at the time of the study, participants self-reported their test results along with their test date in the case of Long-COVID groups. Additional demographic information collected included years of education, handedness, race, and native language. Participants received UCLA IRB-approved telephone screening or online screening for patients followed by an in-person screening (either remote or onsite) for those eligible. Clinical measures assessed on the participants included: the Montreal Cognitive Assessment (MoCA), the 24-item Hamilton Depression Scale (HAMD), the Hamilton Anxiety Scale (HAMA), the Stroke Risk Factor Prediction Chart (CVRF) of the American Heart Association for rating cerebrovascular risk factors, the Cumulative Illness Rating Scale-Geriatric (CIRS-G) used for rating the severity of chronic medical illness in several organ-systems, the Connor-Davidson resilience scale (CDRISC), and the clinical dementia rating (CDR) evaluation used to score the presence or absence of mild dementia. These measures were selected based on the prevalence of symptoms of Long COVID. The HAMA and HAMD are widely used measures to quantify anxiety and depressive symptoms, among the most common neuropsychiatric symptoms of COVID-19. The MoCA is used to assess multiple cognitive domains including memory and executive function and it also used to screen for mild cognitive impairment. The impact of COVID on resilience has also been studied (24). Additionally, the CIRS-G assessed for cognitive symptoms such as brain fog, memory loss, sleep disturbances, etc., while the CVRF was used as a measure of cardiovascular risk and has been established as a hallmark of COVID-19 (25).

### 2.2 Image acquisition and processing

Images were acquired using a Siemens 3T Prisma MRI system at UCLA’s Brain Mapping Center with a 32-channel phased array head coil. Acquisition sequences were identical to the Human Connectome Project Lifespan studies for Aging and Development (https://www.humanconnectome.org). Structural MRIs included T1-weighted (T1w) multi-echo MPRAGE (voxel size=0.8mm isotropic; repetition time (TR)=2500ms; echo time (TE)=1.81:1.79:7.18ms; inversion time (TI)=1000ms; flip angle=8.0o; acquisition time (TA)=8:22 min) and T2-weighted (T2w; voxel size=0.8mm isotropic; TR=3200 ms; TE=564ms; TA=6:35 min) acquisitions with real-time motion correction. Multimodal imaging data were visually inspected and preprocessed with the HCP minimal pipelines (36,37) using the BIDS-App. Utilizing T1w and T2w structural MRI data, PreFreeSurfer, FreeSurfer, and PostFreeSurfer preprocessing streams were used to obtain accurate cortical surface reconstructions for the estimation of cortical gray matter thickness and segmented using the Desikan-Killiany Atlas (26).

Regions of interest (ROIs) were parcellated based on the Desikan atlas separately for each participant after their brains were warped to the atlas space, and the average gray matter volume and cortical thickness was measured for each ROI for each participant. Both gray matter thickness and volumes were analyzed for these regions of interest (ROIs). For the cingulate gyrus, both the individual subdivisions and the combined (adding measures for caudal anterior, isthmus, and posterior cingulate) thickness and volume measures were analyzed.

### 2.3 Statistical analysis

We first compared the Long-COVID and COVID- participants on demographic and clinical characteristics using Fisher’s exact tests for categorical variables and nonparametric Kruskal-Wallis tests for continuous variables. Statistical analysis of gray matter thickness and volumes for group differences between Long-COVID and COVID- participants was conducted using linear regression models with age, sex, and intracranial volumes (ICV) as covariates for the ROIs in R version 4.3.1. Further, fixed effects linear models were used to determine relationships between gray matter structures and clinical variables while controlling for the above covariates. The resulting p-values showing statistical significance were not corrected for multiple comparisons (7 ROIs x 2 hemispheres x 2 measures (GM thickness, GM volume) = 28 measurements).

## 3 Results

### 3.1 Clinical differences between long-COVID and COVID- participants

**Table 1** presents the demographic and clinical characteristics of the study groups. In this sample, participants with Long-COVID were significantly younger than COVID- participants; in addition, they had significantly greater depressive and anxiety symptoms than the COVID- group. The two groups did not differ significantly in their MoCA scores, cardiovascular risk or their cumulative illness ratings (controlling for age).

**Table 1:** Characteristics of study participants.

	Long-COVID	COVID-	p-value*
	N=15	N=8	
Demographics^#^			
Age (years)	41.0 (13.5), 22-62	53.8 (18.3), 20-67	0.05
Sex	7M/8F	3M/5F	1
Education (years)	16.1 (2.0), 13-20	15.8 (1.9), 14-20	0.5
Race			0.3
White	7	4	
Black	0	1	
Asian	2	2	
Other	6	1	
Hispanic ethnicity	7	1	0.2
Mean interval between COVID-19 diagnosis and Imaging (months)	9.3 (6)	-	-
Clinical characteristics^#^			
MoCA (cognitive domains, MCI)	25.7 (2.6), 18-29	24.9 (3.1), 20-27	0.9
HAMD (depression)	11.3 (4.9), 4-19	4.9 (4.4), 1-14	0.01
HAMA (anxiety)	9.6 (4.2), 3-17	4.1 (3.7), 1-12	0.01
CVRF (cardiovascular risk)	4.1 (2.3), 1-9	7.0 (3.9), 2-12	0.2^$^
CIRS-G (chronic medical illness)	4.1 (4.8), 0-15	1.8 (1.4), 0-4	0.1^$^
CDRISC (resilience)	63.8 (12.1), 48-98	67.8 (12.6), 48-88	0.28^$^
CDR (dementia)	N=8 (CDR=0), 0-1	N=7 (CDR=0), 0-1	0.06^##,$^

*p-values determined using Fisher’s exact tests for categorical variables and nonparametric.

Kruskal-Wallis tests for continuous variables.

^#^mean (SD), range for continuous measures; number for categorical variables.

^##^p-value determined using Logistic regression.

^$^controlling for age.

### 3.2 Structural MRI differences between long-COVID and COVID- participants

**Figure 2:**
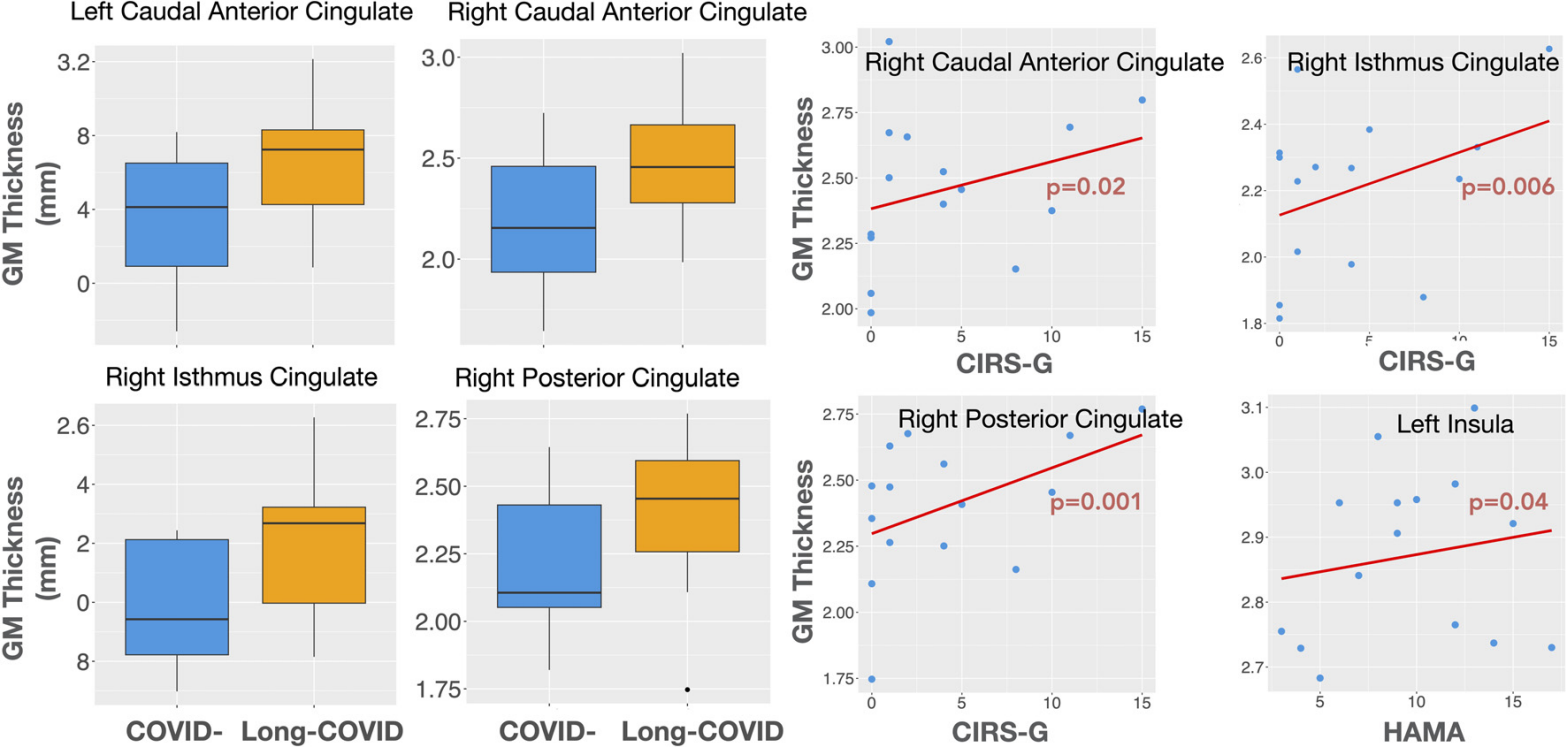
Left: Cortical thickness differences between the Long COVID (Long-COVID) and the COVID- groups for the left and right caudal anterior cingulate and the right isthmus and posterior cingulate ROIs. Right: Structural associations with clinical measures in the Long-COVID group. The right caudal anterior, isthmus, and the posterior cingulate thickness is associated with illness severity (CIRS-G), while the left insular thickness is associated with anxiety (HAMA).

**Figure 3:**
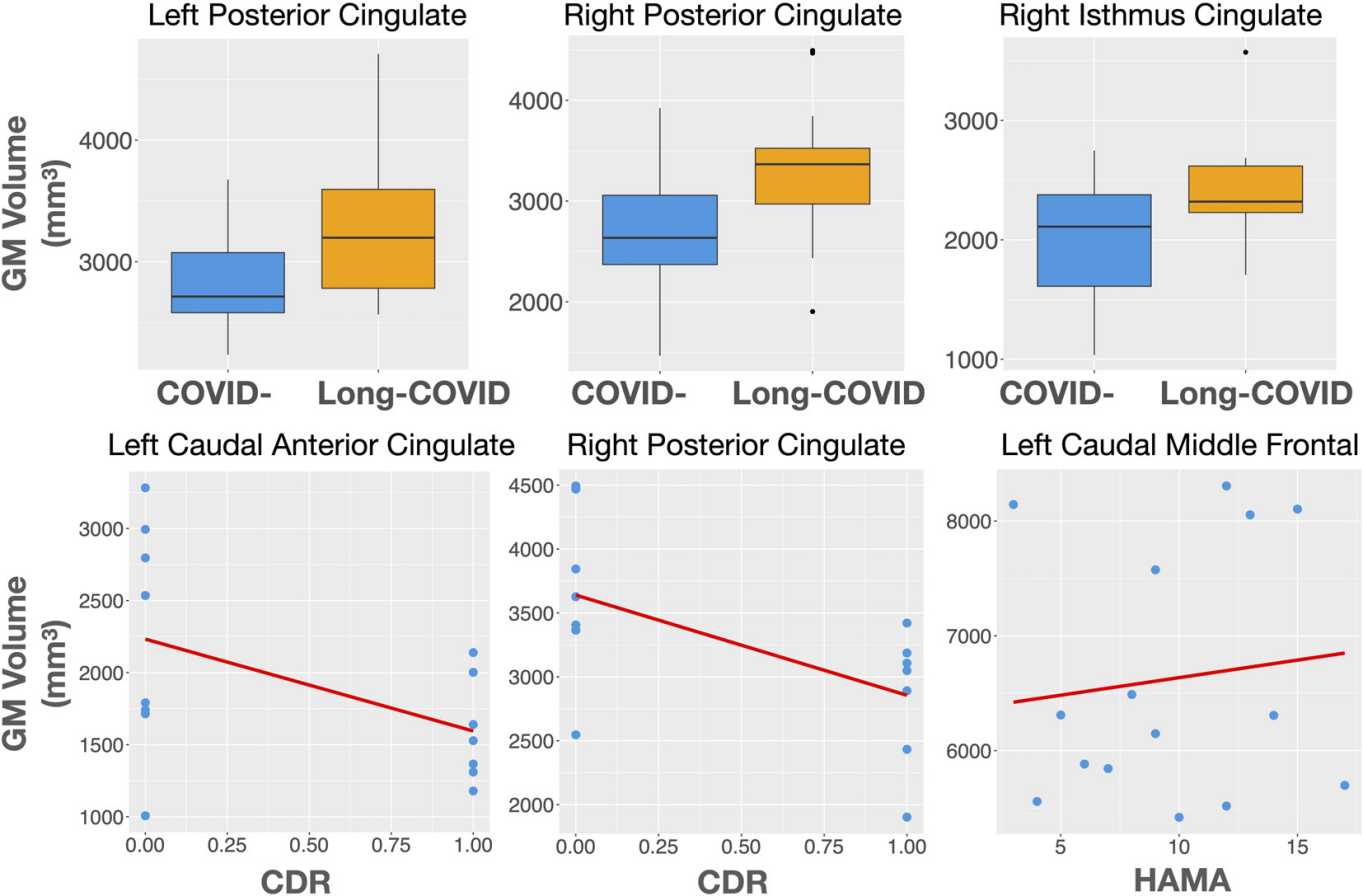
Top row: Gray matter volume differences between the Long-COVID and the COVID- groups for the left and right posterior cingulate and the right isthmus cingulate ROIs. Bottom row: Structural associations with clinical measures in the Long-COVID group. The left caudal anterior and the right posterior cingulate volume is associated with the clinical dementia rating score (CDR) while the left caudal middle frontal gyrus volume is associated with anxiety (HAMA).

The Long-COVID group showed bilaterally higher cortical thickness for the caudal anterior cingulate (left: F=5.75, p=0.03; right: F=5.56, p=0.03), the right posterior cingulate (F=4.69, p=0.04), the right isthmus cingulate (F=8.67, p=0.01), and the right rostral middle frontal gyrus (F=5.65, p=0.03) compared to the COVID- group. Higher gray matter volume was observed in the Long-COVID group in the bilateral posterior cingulate (left, right: F=5.01, p=0.04), and the right isthmus cingulate (F=6.97, p=0.02). Finally, the combined total cingulate showed significant difference in the right hemisphere for both thickness (F=6.06, p=0.02) and volume (F=9.13, p=0.01). **Table 2** lists the groups differences between all the selected ROIs. **Figure 2** (left) shows plots of cortical thickness differences between the Long-COVID and the COVID- groups for the left and right caudal anterior cingulate, the right posterior cingulate, and the right isthmus cingulate. **Figure 3** (top row) shows plots of gray matter volume differences between the Long-COVID and the COVID- groups for the left and right posterior cingulate and the right isthmus cingulate ROIs. None of the differences survived multiple comparisons correction.

**Table 2:** Structural MRI differences between Long-COVID and COVID- participants.

*Brain Region*	*ROI*	*Hemisphere*	*F-value*	*p*	*Sig. (uncorrected)*
			Thickness	Thickness	
			Volume	Volume	
*Dorsolateral* *prefrontal cortex* *(DLPFC)*	Caudal middle frontal gyrus	L	0.02	0.89	
			0.27	0.61	
	Rostral middle frontal gyrus	L	0.28	0.60	
			0.08	0.78	
*Limbic system*	Caudal anterior cingulate	L	5.75	0.03	*
			1.48	0.24	
	Posterior cingulate	L	3.38	0.08	
			5.01	0.04	*
	Isthmus cingulate	L	2.44	0.14	
			0.66	0.43	
	Entorhinal cortex	L	0.97	0.34	
			0.72	0.41	
	Insula	L	3.29	0.09	
			2.93	0.10	
*Dorsolateral* *prefrontal cortex (DLPFC)*	Caudal middle frontal gyrus	R	0.00	0.96	
			0.00	0.95	
	Rostral middle frontal gyrus	R	5.65	0.03	*
			1.40	0.25	
*Limbic system*	Caudal anterior cingulate	R	5.56	0.03	*
			2.40	0.14	
	Posterior cingulate	R	4.69	0.04	*
			5.01	0.04	*
	Isthmus cingulate	R	8.67	0.01	*
			6.97	0.02	*
	Entorhinal cortex	R	0.15	0.71	
			0.27	0.61	
	Insula	R	4.40	0.05	*
			0.25	0.62	

* denotes ROIs with p < 0.05 (uncorrected).

### 3.3 Structural MRI associations with clinical symptoms in the long-COVID group

In the Long-COVID group alone, we found significant (uncorrected for multiple comparisons) positive associations for the ROI gray matter thickness between the right caudal anterior cingulate and CIRS-G (t=2.73, p=0.02, adj.r2 = 0.49), the left and right isthmus cingulate with CIRS-G (left: t=2.35, p=0.04, adj.r2 = 0.33 right: t=3.47, p=0.01, adj.r2 = 0.68), the left and right posterior cingulate and CIRS-G (left: t=2.30, p=0.04, adj.r2 = 0.44 right: t=4.27, p=0.001, adj.r2 = 0.69), and the left insula with HAMA (t=2.39, p=0.04, adj.r2 = 0.25). The left and the right insular cortical thickness was positive associated with CDRISC (t=3.52, p=0.01, adj. r2 = 0.76) and HAMA (t=2.39, p=0.04, adj.r2 = 0.25). Further, the cortical thickness for the left rostral middle frontal gyrus (t=2.87, p=0.02, adj. r2 = 0.39), the left isthmus cingulate (t=3.45, p=0.01, adj. r2 = 0.53), and the right posterior cingulate (t=2.97, p=0.01, adj.r2 = 0.53) showed positive associations with CDRISC.

Finally, the ROI gray matter volumes in the Long-COVID group showed positive associations between the left caudal middle frontal gyrus and HAMA (t=2.31, p=0.04, adj.r2 = 0.31), and a negative association for the left caudal anterior cingulate (t=-3.92, p=0.002, adj.r2 = 0.719818) and the right posterior cingulate (t=-2.60, p=0.03, adj.r2 = 0.48) with CDR, while the right entorhinal cortical volume showed positive associations with CIRS-G (t=2.79, p=0.02, adj.r2 = 0.47) and CDRISC (t=2.45, p=0.03, adj.r2 = 0.41).

**Figure 2** (right) shows the thickness associations between the right caudal anterior cingulate, right posterior cingulate, and the right isthmus cingulate with CIRS-G, and between the left insula and HAMA within the Long-COVID group. The associations for gray matter volume for the left caudal anterior cingulate and the right posterior cingulate with CDR, and between the left caudal middle frontal gyrus and HAMA are shown in **Figure 3** (bottom row).

## 4 Discussion

In a small sample of Long COVID participants, we were able to show differences in cortical thickness between the Long-COVID and the COVID- groups with an increase in gray matter thickness in Long COVID patients compared to the COVID negative group. We were also able to demonstrate cortical thickness associations of the cingulate ROIs and the insula with clinical variables including HAMA and CIRS-G. We note that these effects did not survive multiple comparisons.

Our findings of group differences in structural neural changes contrast with some prior studies. For example, in a large population from the UK Biobank, Douaud et al. (13) show reduced cortical thickness in orbitofrontal cortex and parahippocampal gyrus in patients in a pre-post comparison. Other studies have shown reduced gray matter volume (27) in older adults with COVID-19 (although the participants had also received oxygen therapy when hospitalized) as well as reduced cortical thickness and gray matter volume in patients with varying neurological symptom severity. Our study analyzed cortical thickness, which has a different anatomical interpretation than gray matter volume despite being strongly correlated with each other.

In our sample, within the participants with Long COVID, higher thickness in selected regions of the cingulate was associated with increased severity of chronic medical illness (CIR-G) while higher thickness in the insula was associated with an increased anxiety (HAMA). Additionally, higher gray matter volume in the left caudal middle frontal gyrus was associated with increased anxiety (HAMA), whereas reduced gray matter volume in the Long COVID participants was associated with mild cognitive impairment (CDR). Thus, the more severe the symptoms of illness and anxiety, the greater was the cortical thickness and gray matter volume for the selected regions. These findings are in line with those studies that have found an increase in gray matter thickness (or volume) in participants who are survivors of COVID-19 or those who have Long COVID. For example, our thickness findings trend in the same direction as the volume findings reported by (5, 28, 29) although their findings are longitudinal. Further, it is observed that cortical thickness is more sensitive to aging compared to gray matter volume (30, 31). A COVID-19 imaging study (5) from a VBM (32) analysis have shown an increase in gray matter volumes in the limbic and the olfactory systems and replicates a previous VBM study (29) that also report an increase in gray matter volume. In another pre-post 3 month within-patient, follow up study by 28 (28) COVID-19 patients showed significantly higher bilateral gray matter volumes (GMV) in the olfactory cortex, the hippocampus, insular cortex as well as right cingulate gyrus among other regions. Another study focusing on COVID-19 survivors exhibiting fatigue showed higher gray matter volume in the limbic system and basal ganglia, and the posterior cingulate in particular. Our findings also agree with a recent Long COVID study that showed increase in cortical thickness across multiple groups comprising uninfected healthy participants, COVID-19 survivors, and participants who went on to have Long COVID (with and without cognitive deficits) (22). Particularly they showed greater cortical thickness in the prefrontal and temporal gyri, insula, posterior cingulate, parahippocampal gyrus, and parietal areas.

The disparity in the direction of the effects may be a consequence of the heterogeneous sample of participants with varying levels of symptoms as well as acute- and non-acute presentation of illness. Among several possible reasons, brain tissue swelling due to neuroinflammation and the resulting migration of immune cells (22) or from persistent neurovascular injuries (33) has been suggested to be a putative cause. Alternatively, a compensatory mechanism resulting in neurogenesis and hypertrophy (34) or persistent neuroinflammation (35) may also be considered as explanations for thickness increase.

### 4.1 Study limitations

Limitations of our study include a relatively small and homogeneous sample, two groups not well matched by age with COVID- subjects being older, and the lack of longitudinal follow up. Due to the small sample, the study lacked sufficient power for achieving statistical significance post multiple comparisons correction. Since we did not have neuroimaging data pre- and post-infection, it was not straightforward to determine if the brain changes were transient in nature or long-lasting. While the cardiovascular risk factor was measured in participants, neither markers of cerebrovascular disease nor specific imaging modalities such as CT or angiography were acquired. Similarly, biomarkers of neurodegenerative disease or other comorbidities such as any metabolic/autoimmune were not collected. Another limitation of our study is the lack of peripheral inflammatory markers to investigate the neuroinflammatory correlates of brain imaging data. Besteher et al. showed elevated levels of cytokines including IL-10, IFNγ, and sTREM2 in Long COVID patients, especially in those suffering from cognitive impairment, however, the association between the neuroinflammatory markers and cortical thickness was not presented (22). Since our work focused on structural MRI, investigation of the various brain networks that reflect altered brain function in Long COVID was not possible. Future studies involving functional MRI may help to formulate and resolve network level hypotheses in Long COVID.

Since in our present study, the COVID- subjects were older compared to the Long-COVID population, future studies need to acquire an age-matched large sample with longitudinal follow up and between group comparison to include COVID- subjects with matching neuropsychiatric symptoms and Long-COVID without neuropsychiatric symptoms to further validate our findings. The relationships between brain and behavior examined using structural MRI is subject to limitations of the sample size or functional disruption rather than gross structural abnormalities. In the absence of immunological or markers of neurodegeneration, the non-significant imaging results as well as the associations with behavioral and neuropsychological measures should be interpreted with caution. Additionally, a follow up study may also present opportunities to investigate whether the cortical thickness or gray matter volume increases diminish over time. In summary, our findings should be interpreted in the context of the small sample size of our study compounded with the heterogeneous manifestations of Long COVID. More research is needed along with a comprehensive stratification of the COVID-19 subtypes, which include Long COVID, to explore whether there are compensatory mechanisms of neuroinflammation at play and whether brain imaging in conjunction with neuropsychiatric battery is useful in unraveling these subtypes.

## Data Availability

The raw data supporting the conclusions of this article will be made available by the authors, without undue reservation.
